# Design and Experiment Using Flexible Bumps for Piezoelectric-Driven Hydraulically Amplified Braille Dot Display

**DOI:** 10.3390/mi12070795

**Published:** 2021-07-05

**Authors:** Xiaochao Tian, Yuze Sun, Zhiyao Li, Hu Wang, Zhicong Wang, Haigang Wang, Jinzhi Zhu, Zhigang Yang

**Affiliations:** 1 Institute of Mechanical and Vehicle Engineering, Changchun University, Changchun 130022, China; syz200324@163.com (Y.S.); lizhiyao@ccu.edu.cn (Z.L.); wanghuccdx@163.com (H.W.); w18844010813@163.com (Z.W.); wanghaigangedu@163.com (H.W.); 190101006@mails.ccu.edu.cn (J.Z.); 2 Jilin Provincial Key Laboratory of Human Health Status Identification and Function Enhancement, Changchun 130022, China; 3 College of Mechanical and Aerospace Engineering, Jilin University, Changchun 130025, China; yzg@mail.jlu.edu.cn

**Keywords:** piezoelectric drive, Braille display, fluid drive

## Abstract

This paper describes the design of a piezoelectric-driven hydraulically amplified Braille-flexible bump device that enables the flexible formation of Braille characters. A piezoelectric vibrator is used to excite fluid resonance in a cavity, and displacement is realized by compressing the fluid, allowing Braille character dots to be formed. First, the structural design and working principle of the device, as well as the method used to drive the fluid, are explained. Expressions for the output displacement and amplification ratio of the flexible film and piezoelectric vibrator are then obtained through kinetic analysis of the system unit. Subsequently, the structural parameters that affect the output displacement and the liquid amplification are described. Finally, experimental tests of the system are explained. The results indicate that the output displacement of the contact formed by the flexible film reaches 0.214 mm, satisfying the requirements of the touch sensitivity standard for the blind, when the fluid cavity diameter measures 31 mm and the resonance frequency is 375.4 Hz. The corresponding water discharge is 8.8 mL. This study proves that constructing a Braille bump device in this way is both feasible and effective.

## 1. Introduction

Braille displays, also known as Braille terminals, convert text information from computers into Braille, enabling the blind to interact with computers through tactile human–computer interaction. At present, there are many methods for driving Braille displays. For instance, electromagnetic driving is characterized by high energy consumption, large volumes, and high levels of heat generation [1,2,3,4], while the memory alloy drive type offers fast response speeds but involves large strain levels and requires a specific working environment, which introduces difficulties to actual applications [5,6]. The electrical stimulation approach involves direct contact with the skin, which can be corrosive and causes a tingling sensation [7,8]. Braille point display devices driven by electroactive polymers have low efficiency and low response speeds and are prone to fatigue and aging [9,10,11,12]; other drive methods have similar limitations [13,14]. Therefore, in order to be able to make up for the above shortcomings and achieve a more environmentally friendly and efficient drive, this paper describes a device that produces a Braille dot display effect using piezo-electric oscillator vibration. This vibration excites the liquid in a cavity, generating system resonance and compressing the fluid to drive a flexible film. It is this film that forms Braille bumps and realizes the dot display function. Compared with the above-mentioned traditional driving method, piezoelectric-driven braille display has low power consumption, does not generate heat, and has no magnetic interference. Piezoelectric-driven Braille displays involve the application of positive and negative voltages to induce a piezoelectric vibrator to bend and deform, whereby the contact rod fixed on the piezoelectric material moves up or down. This motion forms bumps or falling cycle switching, producing refreshable Braille characters [15,16,17]. 

## 2. Structural Composition and Working Principle

### 2.1. Material and Structure Composition

**Figure 1:**
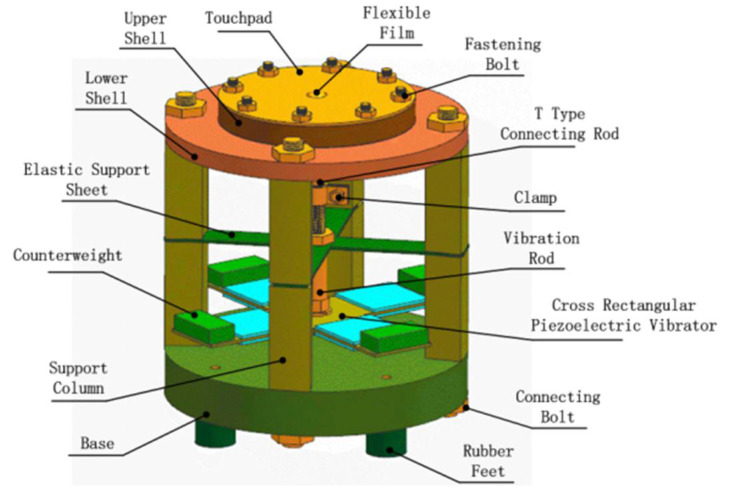
Structural illustration of flexible convex device.

The structure of the proposed flexible Braille bump device is shown in Figure 1. The device is mainly composed of a cross-shaped rectangular piezoelectric vibrator, counterweight, vibration transmission rod, elastic support sheet, T-shaped connecting rod, elastic sheet, clamp, lower shell, sealing ring, upper shell, flexible film, and touchpad.

The touchpad, upper shell, and lower shell are all made of acrylic board. There is a groove on the upper end surface of the upper shell and the inside of the lower shell, which is used to place the O-ring seal. An infusion hole is designed on the side of the upper shell, which has the functions of injecting liquid and exhausting air. The flexible film is made of latex, which has good flexibility and elasticity. The flexible film is placed on the surface of the upper shell and pressed into the groove with an O-ring; then the touchpad is pressed on the flexible film, and, under the action of hydraulic pressure, the flexible film will deform to form braille character dots for blind people to touch and read. The fluid medium selected here is water, which has the advantages of low compressibility, good fluidity, no pollution, and easy access. The base bracket is the connection structure of the various parts of the whole assembly. It is composed of rubber feet, base, and support columns. The base bracket is made of cast iron material, which has the characteristics of good stability and easy processing.

### 2.2. Working Principle

The steps of making a cross-shaped rectangular piezoelectric vibrator are to first glue eight rectangular piezoelectric ceramic sheets on both sides of the “cross”-shaped copper substrate with epoxy resin and then stick the weight mass on the edge of the “cross” copper substrate. Compared with the traditional circular piezoelectric vibrator, the cross-shaped structure has a symmetrical structure that can provide a stable power drive for the entire device.

An alternating signal is applied to the piezoelectric vibrator, which generates a reciprocating bending deformation, with the weight strengthening the vibration energy. The transmission rod transfers the vibration energy to the elastic sheet, although this energy is negligible due to the liquid’s minimal compressibility. When the external input frequency is close to or consistent with the system’s natural frequency, the system resonates. At this time, the amplitude of the excitation device reaches a maximum. Under the inertia of the liquid medium, the displacement at the center of the flexible film is further magnified. The protruding flexible film thus forms a soft-touch, non-sharp Braille character bump.

## 3. Analysis and Testing of Amplifying Unit

### 3.1. Theoretical Analysis

**Figure 2:**
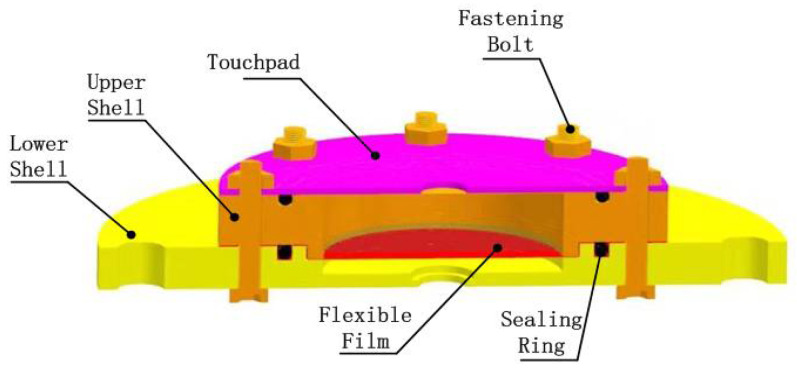
Schematic diagram of the fluid chamber structure.

The fluid chamber structure of the device is shown in Figure 2. The main structure is composed of a touch panel, sealing ring, flexible film, upper casing, and lower casing. There are grooves on the upper shell’s upper-end surface and the inside of the lower shell, allowing the placement of an O-shaped sealing ring, and there are infusion holes on the side for liquid injection and exhaust. The flexible film is placed on the upper shell’s inner surface, pressed into the groove with an O-shaped sealing ring. The touchpad is then pressed onto the flexible film, which protrudes to form flexible Braille character dots under the action of hydraulic force. 

**Figure 3:**
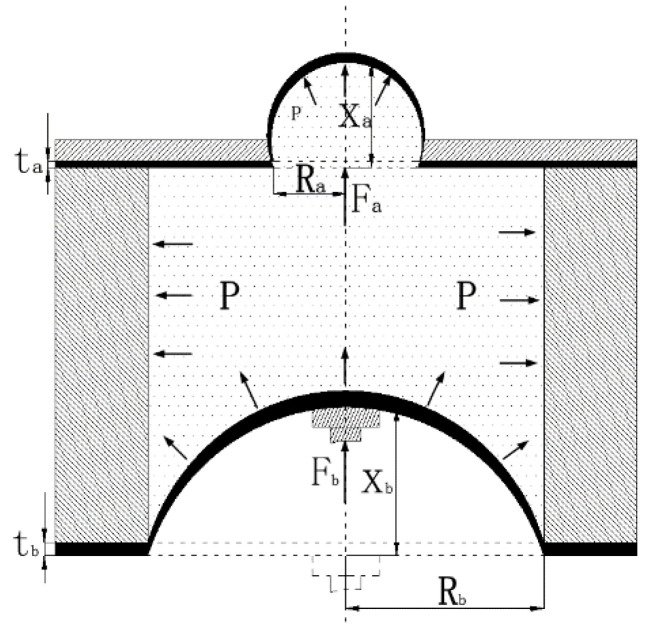
Simplified model of hydraulic amplifier unit.

A simplified model of the hydraulic amplifier unit is shown in Figure 3. In the figure, *R*_a_ is the radius of the flexible film, *R*_b_ is the radius of the elastic sheet, *X*_a_ is the output displacement of the flexible film, *X*_b_ is the displacement of the elastic sheet, *t*_a_ is the thickness of the flexible film, *t*_b_ is the thickness of the elastic sheet, *F*_a_ is the force received by the flexible film, *F*_b_ is the force received by the elastic sheet, and *P* is the pressure in the cavity, which is equal throughout.

When the cavity is filled with liquid, the liquid can be regarded as being replaced by a rigid body of equal weight and defined as an “equivalent rigid body.” At this time, the liquid-filled rigid body system with infinite degrees of freedom can be transformed into multi-rigid body motion with a finite number of degrees of freedom.

The dynamic differential equation is established as:

(1) $$\left[\begin{array}{cc} m_{11} & m_{12}\\ m_{12} & m_{22} \end{array}\right]\left\{\begin{array}{c} {\ddot{x}}_{1}\\ {\ddot{x}}_{2} \end{array}\right\}+\left[\begin{array}{cc} c_{11} & c_{12}\\ c_{12} & c_{22} \end{array}\right]\left\{\begin{array}{c} {\dot{x}}_{1}\\ {\dot{x}}_{2} \end{array}\right\}+\left[\begin{array}{cc} k_{11} & k_{12}\\ k_{12} & k_{22} \end{array}\right]\left\{\begin{array}{c} x_{1}\\ x_{2} \end{array}\right\}=\left\{\begin{array}{c} F_{1}\\ F_{2} \end{array}\right\}$$

The simple harmonic force of the external excitation is

(2) $$F_{j}\left(t\right)=F_{j0}e^{i\omega t},\;j=1,2.$$

The steady-state solution of Equation (1) is

(3) $$F_{j}\left(t\right)=F_{j0}e^{i\omega t},\;j=1,2.$$

Substituting Equations (2) and (3) into Equation (1) gives

(4) $$\left[\begin{array}{cc} -\omega^{2}m_{11}+i\omega c_{11}+k_{11} & -\omega^{2}m_{12}+i\omega c_{12}+k_{12}\\ -\omega^{2}m_{12}+i\omega c_{12}+k_{12} & -\omega^{2}m_{22}+i\omega c_{22}+k_{22} \end{array}\right]\left\{\begin{array}{c} X_{1}\\ X_{2} \end{array}\right\}=\left\{\begin{array}{c} F_{a}\\ F_{b} \end{array}\right\}.$$

The mechanical impedance *Z*_rs_ is

(5) $$Z_{rs}\left(iw\right)=-w^{2}m_{rs}+iwc_{rs}+k_{rs},r,s=1,2.$$

We can write Equation (4) as

(6) [Z(iw)]X=F

where

(7) $$\begin{array}{c} \left[Z\left(iw\right)\right]=F_{0}\left[\begin{array}{cc} Z_{11}\left(iw\right) & Z_{12}\left(iw\right)\\ Z_{12}\left(iw\right) & Z_{22}\left(iw\right) \end{array}\right]=Impendance\;Matrix\\ X=\left\{\begin{array}{c} X_{1}\\ X_{2} \end{array}\right\},\;F=\left\{\begin{array}{c} F_{a}\\ F_{b} \end{array}\right\} \end{array}$$

The solution of Equation (6) is written as follows:

(8) $$X={\left[Z\left(iw\right)\right]}^{-1}F$$

where the inverse matrix of the impedance matrix is:

(9) $${\left[Z\left(iw\right)\right]}^{-1}=\frac{1}{Z_{11}\left(iw\right)Z_{22}\left(iw\right)-Z_{12}^{2}\left(iw\right)}\left[\begin{array}{cc} Z_{22}\left(iw\right) & -Z_{12}\left(iw\right)\\ -Z_{12}\left(iw\right) & Z_{11}\left(iw\right) \end{array}\right]$$

From Equations (8) and (9), the following solutions can be obtained:

(10) $$\begin{array}{c} X_{1}\left(iw\right)=\frac{Z_{22}\left(iw\right)F_{a}-Z_{12}\left(iw\right)F_{b}}{Z_{11}\left(iw\right)Z_{22}\left(iw\right)-Z_{12}^{2}\left(iw\right)}\\ X_{1}\left(iw\right)=\frac{-Z_{12\left(i\omega \right)}F_{a}+Z_{11\left(i\omega \right)}F_{b}}{Z_{11}\left(iw\right)Z_{22}\left(iw\right)-Z_{12}^{2}\left(iw\right)} \end{array}\}$$

**Figure 4:**
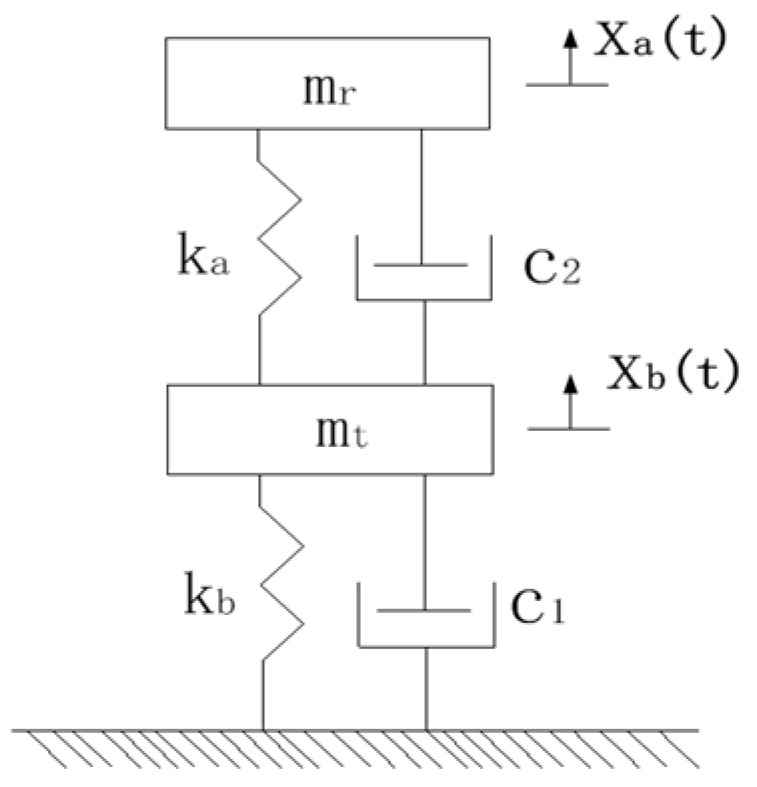
Equivalent spring mass model.

The equivalent spring mass model of the system is shown in Figure 4, where *k*_b_ and *m*_t_ represent the equivalent stiffness and equivalent mass of the elastic sheet. As the equivalent mass of the flexible film and the equivalent mass of the fluid medium have the same laws of motion, the flexible film’s equivalent mass can be attributed to the fluid medium. *m*_r_ represents the equivalent mass of both, and *k*_a_ represents the corresponding equivalent stiffness.

The motion of the equivalent mass in the system can be described by the coordinates *x*_a_(t) and *x*_b_(t). *x*_a_(t) represents the fluid mass and the equivalent quality of the flexible film at time *t*, *m*_r_ denotes the displacement of the center of mass from the equilibrium position, *x*_b_(t) denotes the equivalent mass of the elastic sheet at time *t*, and *m*_t_ indicates the displacement of the center of mass from the equilibrium position. The differential equations of motion of the system can be written as:

(11) $$\left[\begin{array}{cc} m_{t} & 0\\ 0 & m_{t} \end{array}\right]\left\{\begin{array}{c} {\ddot{x}}_{1}\\ {\ddot{x}}_{2} \end{array}\right\}+\left[\begin{array}{cc} c_{1}+c_{2} & -c_{2}\\ -c_{2} & c_{2} \end{array}\right]\left\{\begin{array}{c} {\dot{x}}_{1}\\ {\dot{x}}_{2} \end{array}\right\}+\left[\begin{array}{cc} 2k_{b} & -k_{b}\\ -k_{b} & k_{z}+k_{a} \end{array}\right]\left\{\begin{array}{c} x_{1}\\ x_{2} \end{array}\right\}=\;\left\{\begin{array}{c} bKd_{33}V\\ 0 \end{array}\right\}$$

where *b* is the conversion matrix, *K = A/SEt* is the stiffness of the transducer when it is short-circuited (*V* = 0), which represents the electromechanical coupling coefficient, and *V* represents the voltage. Let the solution be:

(12) $$x_{j}\left(t\right)=X_{j}e^{iwt},\;\;\;\;\;j=1,2$$

From Equation (5), we have

(13) $$\begin{array}{c} Z_{11}\left(iw\right)=-m_{z}w^{2}+\left(c_{1}+c_{2}\right)w+2k_{z}\\ Z_{22}\left(iw\right)=-m_{s}w^{2}+c_{2}w+k_{z}+k_{m}\\ Z_{12}\left(iw\right)=-c_{2}w-k_{z} \end{array}\}$$

Therefore, from Equation (10), we obtain:

(14) $$X_{b}\left(w\right)=\frac{\left(c_{2}\omega +k_{b}\right)bKd_{33}V}{\left[-m_{t}\omega^{2}+\left(c_{1}+c_{2}\right)\omega +2k_{b}\right]\left[-m_{r}\omega^{2}+c_{2}\omega +k_{a}+k_{b}\right]-{\left(c_{2}\omega +k_{b}\right)}^{2}}$$

(15) $$X_{a}\left(w\right)=\frac{\left(c_{2}\omega +k_{b}\right)bKd_{33}V}{\left[-m_{t}\omega^{2}+\left(c_{1}+c_{2}\right)\omega +2k_{b}\right]\left[-m_{r}\omega^{2}+c_{2}\omega +k_{a}+k_{b}\right]-{\left(c_{2}\omega +k_{b}\right)}^{2}}$$

Here, *X*_a_(*ω*) and *X*_b_(*ω*) represent the vibration amplitudes of the equivalent mass center of the fluid medium and the elastic sheet, respectively, and *λ* represents the displacement amplification ratio, given by:

(16) $$\lambda =\frac{\left|X_{b}\left(\omega \right)\right|}{\left|X_{a}\left(\omega \right)\right|}=\frac{\left|c_{2}\omega +k_{b}\right|}{\left|-m_{r}\omega^{2}+c_{2}\omega +k_{b}+k_{a}\right|}$$

where [18]:

$$k_{b}=\frac{F_{b}R_{b}^{2}}{16\pi D_{b}}=\frac{3F_{b}R_{b}^{2}\left(1-v_{b}^{2}\right)}{4E_{b}\pi t_{b}^{3}}$$

$$k_{a}=\frac{F_{a}R_{a}^{2}}{16\pi D_{a}}=\frac{3F_{a}R_{a}^{2}\left(1-v_{a}^{2}\right)}{4E_{a}\pi t_{a}^{3}}$$

In these expressions, *R*_a_ and *R*_b_ denote the radii of the flexible film and the elastic sheet, respectively; *E*_a_ and *E*_b_ are the elastic moduli of the flexible film and the elastic sheet, respectively; and *v*_a_ and *v*_b_ are the Poisson’s ratios of the flexible film and the elastic sheet, respectively, where:

$$D_{b}=\frac{E_{b}t_{b}^{3}}{12\left(1-v_{b}^{2}\right)}$$

$$D_{a}=\frac{E_{a}t_{a}^{3}}{12\left(1-v_{a}^{2}\right)}$$

$$F_{a}=P\cdot S_{a}=\frac{F_{b}\cdot S_{a}}{S_{b}}$$

From the above analysis, under the assumption that the materials of the elastic sheet and the flexible film are known, several primary factors affect the output displacement of the hydraulic amplification unit (i.e., the thickness *t*_a_ and *t*_b_ of the flexible film, the elastic sheet, and the radii *R*_a_ and *R*_b_). The structural and material specifications of the flexible film and the elastic sheet are listed in Table 1. The hydraulic amplification unit achieves a theoretical magnification factor of 3.92.

### 3.2. Output Displacement Test

**Figure 5:**
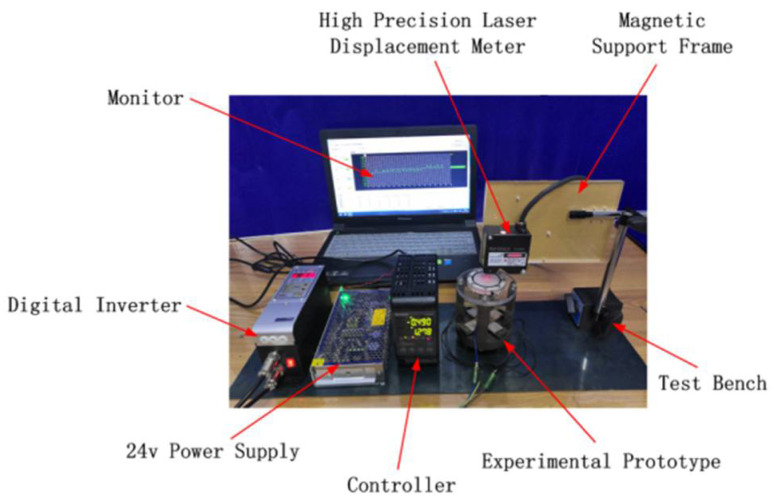
Displacement measurement device.

**Figure 6:**
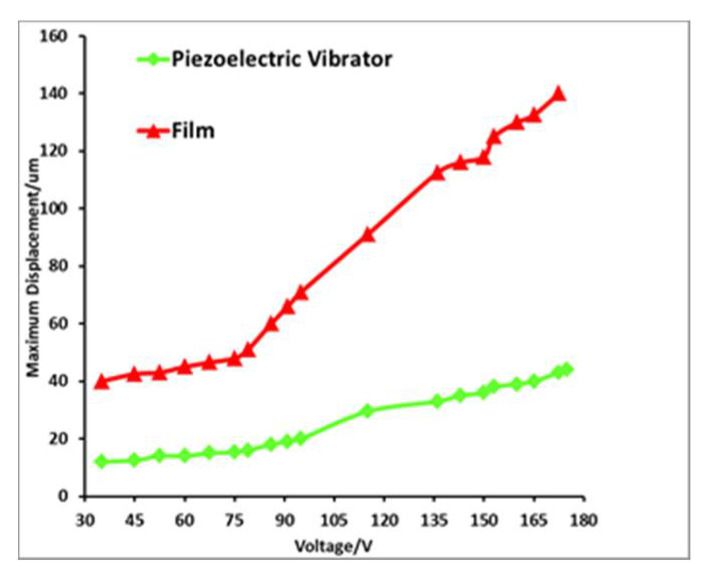
Maximum displacement curve of piezoelectric vibrator and membrane.

The displacement of the piezoelectric oscillator with the flexible film was tested at various voltages using a laser micrometer. The test setup is presented in Figure 5. The results are shown in Figure 6. As can be observed, the hydraulic amplification system results in a significant amplification of the piezoelectric oscillator. When the driving voltage is 150 V, the displacement of the piezoelectric vibrator is 35 μm, and the displacement of the corresponding flexible film is 115 μm. The magnification factor is 3.85, slightly smaller than the theoretical value. This is because the liquid’s compressibility and processing accuracy cause the magnification test value to be slightly lower than the theoretical value.

## 4. Testing and Analysis

### 4.1. Instruments and Parameters

Experimental testing devices include monitors, test prototypes, frequency conversion controllers, high-precision laser displacement meters, sensor support frames, test benches, and 24 V power supplies. Additional tools such as syringes and measuring cups were used for the production of four test scenarios. The displacement and deformation of the flexible film was measured by changing the driving voltage and frequency in order to find the ideal prototype parameters. The basic experimental parameters of the four groups of prototypes are shown in Table 2.

### 4.2. Influence of Driving Voltage on Output Displacement of Flexible Film

**Figure 7:**
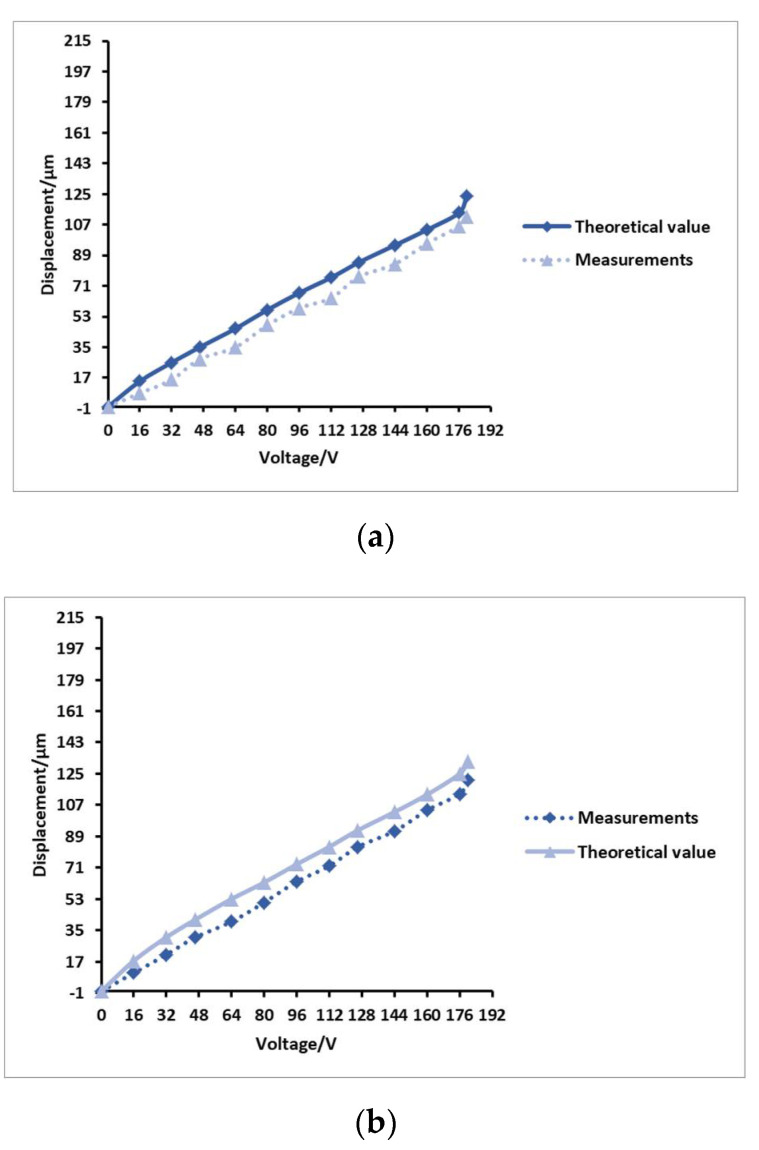

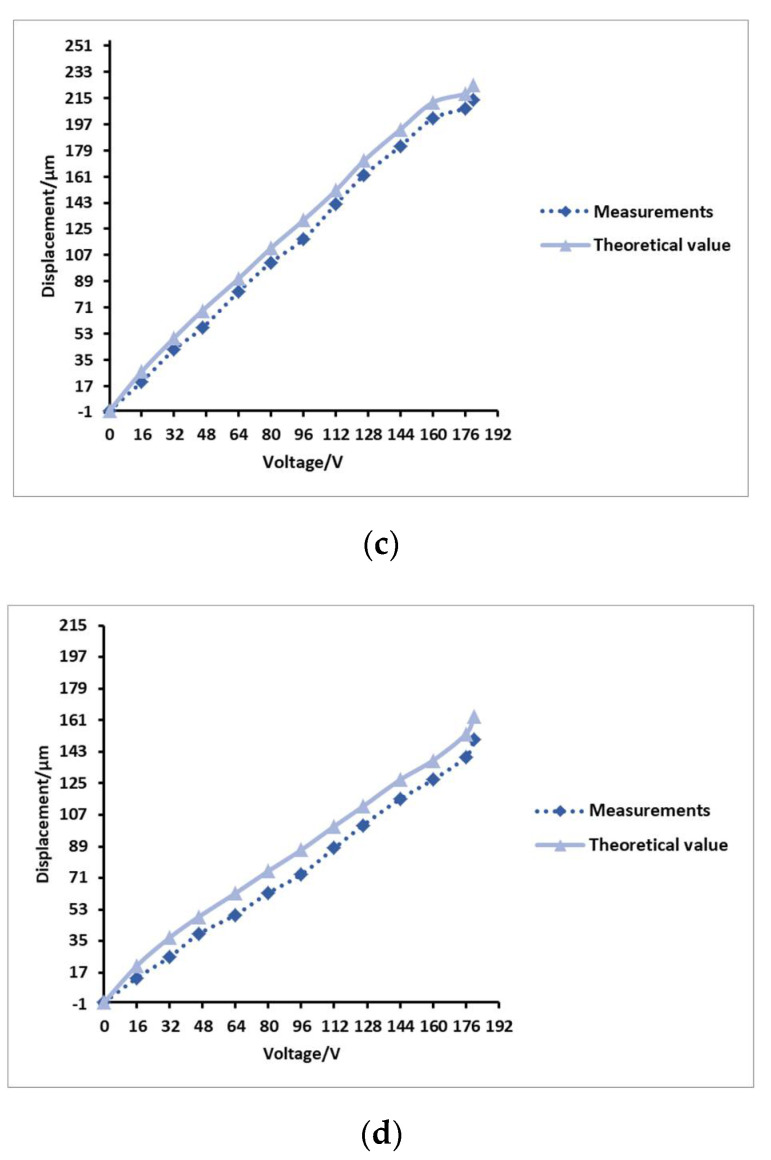
Comparative diagram of four sets of prototype drive voltages and flexible film displacement. (**a**) The relationship between the driving voltage of the No. 1 prototype and the displacement of the flexible film; (**b**) the relationship between the driving voltage of the No. 2 prototype and the displacement of the flexible film; (**c**) the relationship between the driving voltage of the No. 3 prototype and the displacement of the flexible film; (**d**) the relationship between the driving voltage of the No. 4 prototype and the displacement of the flexible film.

Theoretical analysis found that a higher driving voltage supplies greater vibration energy to the piezoelectric vibrator. This enhances the amplitude of the elastic sheet, resulting in greater output displacement by the hydraulic amplifier unit. Actual measurements were carried out on the four groups of prototypes. In the case of the same driving frequency, the theoretical value and measured value of the flexible film relative to the driving voltage were compared, and the curve test results are shown in Figure 7.

It can be seen from the four sets of groups in the figure that the output displacement of the flexible film is proportional to the driving voltage of the piezoelectric vibrator, meaning that a higher voltage produces a greater output displacement in the flexible film. When the device reaches the resonance frequency, the displacement will increase significantly, which also shows that increasing the voltage by an appropriate amount can increase the flexible film’s output displacement, meaning that the Braille bumps are more apparent. However, an excessively high voltage will reduce the service life of the piezoelectric vibrator, causing the system to work abnormally. Therefore, the driving voltage of the piezoelectric vibrator must be set to an appropriate value. Obviously, under the same driving voltage, the output displacement of the flexible film of the No. 3 sample is the largest, and the bumps are more obvious. It can be concluded that when the prototype parameter fluid cavity diameter is 31 mm, the fluid cavity height is 6 mm, and the water filling volume is 8.8 mL, the structure size is ideal.

### 4.3. Influence of Driving Frequency on Output Displacement of Flexible Film

**Figure 8:**
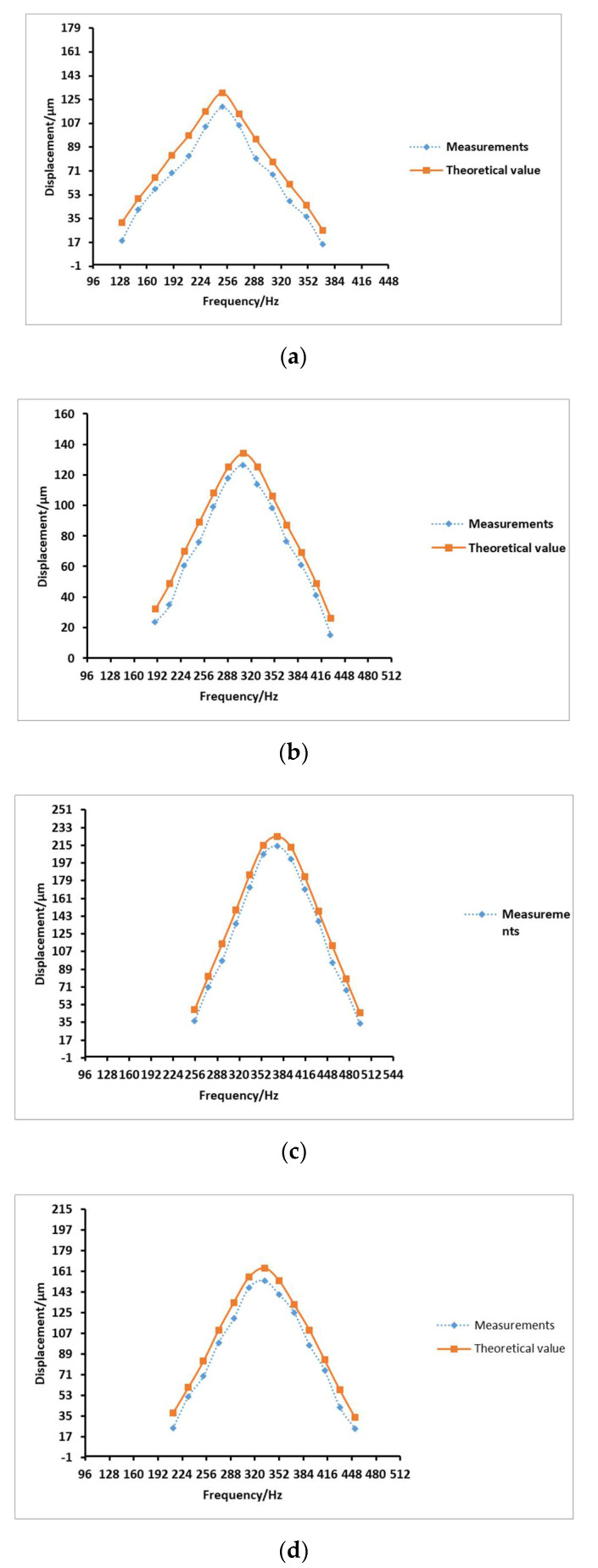
Curve of driving frequency versus displacement of the flexible membrane of the four sets of prototypes. (**a**) The driving frequency and displacement curve of flexible membrane of prototype 1; (**b**) the driving frequency and displacement curve of flexible membrane of prototype 2; (**c**) the driving frequency and displacement curve of flexible membrane of prototype 3; (**d**) the driving frequency and displacement curve of flexible membrane of prototype 4.

The voltage was set to 180 V, and the driving frequency was varied to test the influence of different driving frequencies on the system output. Compared with the theoretical values, the comparison results are shown in Figure 8.

It can be seen from the four sets of Figure 8 that the displacement of the flexible film increases and then decreases as the driving frequency increases. Overall, compared to the other three groups of prototypes, No. 3 has the best output effect at the resonance frequency. The greatest displacement of the flexible film, and thus the optimal output effect, occurs at the resonance frequency of prototype group 3, which is 375.4 Hz. The corresponding displacement of the flexible film is 0.214 mm.

### 4.4. Influence of Fluid Cavity Diameter on Output Displacement of Flexible Film

**Figure 9:**
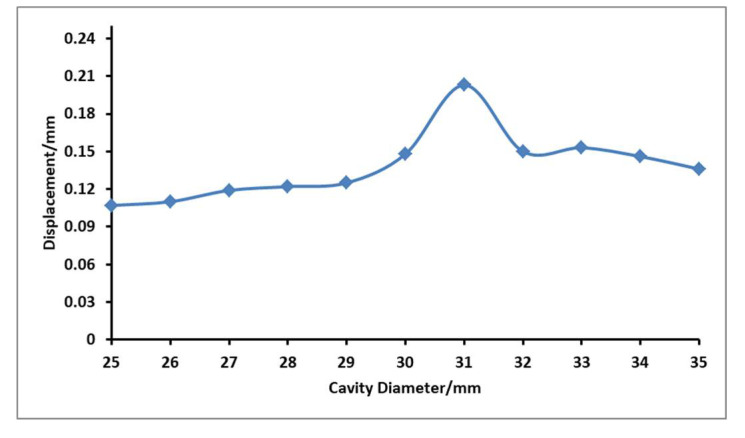
Curves of the relationship between different cavity diameters and flexible film displacement.

When the voltage is 180 V and the resonance frequency is 375.4 Hz, to ensure the same height of fluid cavity, the influence of different cavity diameter on the output displacement of flexible film was explored, and the measurement curve is shown in Figure 9.

It can be seen from the figure that with the increase in the cavity diameter, the overall law of the flexible film is obvious that the displacement first increases and then decreases with the increase of cavity diameter. When the cavity diameter is 31 mm, the effect of point display is very obvious, and the output amplitude can reach 0.203 mm.

### 4.5. Influence of Fluid Cavity Height on Output Displacement of Flexible Film

**Figure 10:**
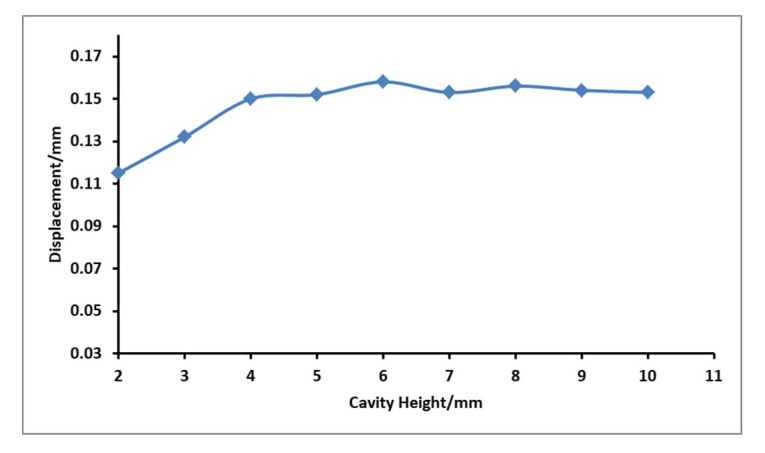
Relationship between different cavity height and flexible film displacement.

When the voltage is 180 V, the resonant frequency is 375.4 Hz, the liquid cavity diameter is 31 mm, and the tension of the flexible film is the same, the influence of the cavity height on the output displacement of the flexible film is explored, and the test curve is shown in Figure 10.

It can be seen from the figure that under the premise that the cavity diameter is the same as the initial conditions, the height of the cavity has little effect on the point display effect, basically no effect. When the diameter is 4−10 mm, the output displacement can reach more than 0.15 mm, and the point display effect is more significant.

## 5. Conclusions

A flexible Braille bump device based on piezoelectric-driven liquid amplification has been designed. The piezoelectric-driven fluid resonates to form Braille characters. The system has been theoretically analyzed, and the displacement value of the flexible film bump was found to be related to the structural parameters of the system. When the fluid cavity has a diameter of 31 mm, height of 6 mm, a resonance frequency of 375.4 Hz, and a flushing volume of 8.8 mL, the output displacement of the character bumps formed by the flexible film is 0.214 mm, which meets the requirements of the touch sensitivity standard for the blind. This study verifies that it is feasible and practical to construct a Braille bump device in this way.

## Figures and Tables

**Table 1:** Structural parameters of flexible film and elastic sheet.

Material	*E* /Gpa	*R* /mm	*T* /mm	*M* /g	*K* N/m	*V*	*F* /N	*X* /μm	*C*
**Flexible** **Film(a)**	7.84 × 10^−3^	7	0.5	3	111	0.47	0.58	115	1
**Elastic** **Sheet(b)**	128	13	0.4	1.5	241,597	0.35	2	397	0.008

**Table 2:** Parameter list of four experimental prototypes.

Group r	Fluid Cavity Diameter	Fluid Cavity Height	Water Filling
**First**	27 mm	4 mm	7.5 mL
**Second**	29 mm	5 mm	8.2 mL
**Third**	31 mm	6 mm	8.8 mL
**Fourth**	32 mm	7 mm	9.3 mL

## Data Availability

The data that support the findings of this study are available from the corresponding author upon reasonable request.
